# Mesenchymal stromal cell treatment attenuates repetitive mild traumatic brain injury-induced persistent cognitive deficits via suppressing ferroptosis

**DOI:** 10.1186/s12974-022-02550-7

**Published:** 2022-07-14

**Authors:** Dong Wang, Shishuang Zhang, Xintong Ge, Zhenyu Yin, Meimei Li, Mengtian Guo, Tianpeng Hu, Zhaoli Han, Xiaodong Kong, Dai Li, Jing Zhao, Lu Wang, Qiang Liu, Fanglian Chen, Ping Lei

**Affiliations:** 1 Haihe Laboratory of Cell Ecosystem, Department of Geriatrics, Tianjin Medical University General Hospital, Tianjin, China; 2 Tianjin Geriatrics Institute, Tianjin Medical University General Hospital, Tianjin, China; 3 Tianjin Neurological Institute, Tianjin Medical University General Hospital, Tianjin, China

**Keywords:** Repetitive mild traumatic brain injury, Mesenchymal stromal cells, Ferroptosis, Glutathione peroxidase 4

## Abstract

The incidence of repetitive mild traumatic brain injury (rmTBI), one of the main risk factors for predicting neurodegenerative disorders, is increasing; however, its underlying mechanism remains unclear. As suggested by several studies, ferroptosis is possibly related to TBI pathophysiology, but its effect on rmTBI is rarely studied. Mesenchymal stromal cells (MSCs), the most studied experimental cells in stem cell therapy, exert many beneficial effects on diseases of the central nervous system, yet evidence regarding the role of MSCs in ferroptosis and post-rmTBI neurodegeneration is unavailable. Our study showed that rmTBI resulted in time-dependent alterations in ferroptosis-related biomarker levels, such as abnormal iron metabolism, glutathione peroxidase (GPx) inactivation, decrease in GPx4 levels, and increase in lipid peroxidation. Furthermore, MSC treatment markedly decreased the aforementioned rmTBI-mediated alterations, neuronal damage, pathological protein deposition, and improved cognitive function compared with vehicle control. Similarly, liproxstatin-1, a ferroptosis inhibitor, showed similar effects. Collectively, based on the above observations, MSCs ameliorate cognitive impairment following rmTBI, partially via suppressing ferroptosis, which could be a therapeutic target for rmTBI.

## Background

A traumatic cerebral concussion can induce mild traumatic brain injury (mTBI), accounting for 75% of all reported cases of head injury, and mTBI can cause various debilitating symptoms, including headaches, irritability, fatigue, attention deficits, sleep disturbances, depression, and anxiety [1, 2]. However, the incidence of mTBI is grossly underestimated since most individuals completely recover within the first days to weeks after injury, preventing them from seeking medical treatment [3]. People with sustained single mTBI have an increased risk of developing repetitive mTBI (rmTBI) [4]. RmTBI has been identified as the main risk factor for neurodegenerative disorders, including Parkinson’s disease (PD), Alzheimer’s disease (AD), chronic traumatic encephalopathy, frontotemporal dementia, and is of particularly high relevance to military personnel, athletes, elderly and people with mobility impairments [5]. The incidence of rmTBI is increasing; however, little is known about its underlying mechanism, seriously affecting the progress of research on therapies for rmTBI and the prognosis of the disease. During the pathological process of rmTBI, different forms of cell death, such as apoptosis, autophagy, and necrosis, have been reported [6]. We wondered whether completely new forms of cell death exist during this process.

Ferroptosis, a recently discovered programmed cell death form caused by lipid damage under iron catalysis, is probably one of the most ancient and widespread forms of cell death [7–9]. Ferroptosis shows a significant difference compared with additional cell death pathways, in which ATP consumption, mitochondrial reactive oxygen species (ROS) production (necroptosis mediators), caspases (apoptosis and pyroptosis mediators), and increased levels of intracellular Ca^2+^ or Bax/Bak (necessary outer mitochondrial membrane permeabilization mediators) levels are not involved [10]. The main hallmarks of ferroptosis are mitochondrial shrinkage, iron/lipid ROS accumulation, and glutathione peroxidase 4 (GPx4) inactivation [11, 12].

Iron, an important component of hemoglobin and many enzymes, plays an important role in different basic metabolic processes. However, its aberrant homeostasis is related to ferroptosis, which can induce central nervous system (CNS) pathology [13]. Multiple proteins maintain iron homeostasis in cells, regulation of transferrin receptor-1 (Tfr1) maintains iron consumption, and ferroportin (Fpn) regulates iron export [14, 15]. GPx4, a selenoprotein glutathione peroxidase, plays a crucial role in the regulation of ferroptosis, which exhibits a direct detoxification effect on hydroperoxides in membrane lipids; thus, decreasing membrane functional impairment while suppressing the production of reactive products, such as 4-hydroxynonenal (4-HNE), obtained from lipid peroxidation [16]. The failure of GPx4 action will lead metabolic disturbance of lipid oxide within cells under iron ion catalysis, which will cause ROS accumulation on membrane lipids, redox imbalance in cells, and ferroptosis induction [17]. Erastin and glutamate can trigger ferroptosis [18], and liproxstatin-1 (Lip-1) can suppress it [19]. Ferroptosis is related to different disorders such as cancer, lung ischemia–reperfusion injury, brain stroke, ischemic heart disease, intracerebral hemorrhage, and TBI [17]. Ferroptosis has been suggested to directly or indirectly contribute to a variety of persistent effects after TBI, including cognitive deficits, neurodegeneration, post-trauma epilepsy, and psychiatric symptoms [13]. Taking into account the potentially significant effect of ferroptosis on the course of persistent impaired cognition after rmTBI, in this study, we explored the occurrence of ferroptosis after rmTBI and developed novel methods to suppress ferroptosis-associated cell death to attenuate tissue damage from rmTBI and improve persistent cognitive function.

Stem cells (SCs) have attracted a great deal of attention as a potent candidate in tissue engineering and regenerative medicine. Since their initial test in humans as a cell pharmaceutical agent in 1995, mesenchymal stromal cells (MSCs) have been extensively used in clinical experiments due to their high self-renewal capacity, multidirectional differentiation potential, extremely low immunogenicity, and remarkable clinical safety [20–22]. MSCs can produce neurotrophic growth factors, regulate immunity while helping wound healing, and promote nerve cell health through mitochondrial donation [23]. Therefore, MSCs can be a possible way to treat acute damage or progressive CNS degenerative disorders [24–26], including TBI, AD, PD, and multiple system atrophy [23]. Although MSCs have therapeutic potential in many disorders, it is unclear whether MSCs inhibit ferroptosis and reduce neurodegeneration after rmTBI.

In the present study, we focus on investigating the appearance of ferroptosis following rmTBI and the role of the MSC intervention in inhibiting ferroptosis and improving persistent cognitive function in rmTBI.

## Methods

### Animals

We obtained a total of 300 adult male C57BL/6J mice (8–12 weeks old, weight 22–24 g) from the Chinese Academy of Military Sciences (Beijing, China). Each experiment was performed according to the NIH Guide for the Care and Use of Laboratory Animals. The Animal Care and Use Committee of Tianjin Medical University approved our study protocols. All animals were raised and adaptively fed for one week before further experiments.

### rmTBI mouse model induced by controlled cortical impact (CCI)

To study whether ferroptosis occurs after rmTBI, we used an as-reported rmTBI model [27–29]. A molded acrylic cast was designed to fix the mice and provided 3.0 mm of space below their heads for acceleration and deceleration beneath the point of impact. The mice were anesthetized with 4.6% isoflurane and secured in the prone position to acrylic plaster with surgical tape across their shoulders. Following head shaving, we attached a self-designed standard manufacturing concave metal disc (diameter = 3 mm) to the skull caudally to the bregma as a helmet to transmit the hitting power to the entire brain, resulting in a mild diffused brain injury, but not focal brain injury, thus exerting the pathological change similar to clinical rmTBI. In addition, a scalp incision was not made in order to better simulate the injured procedure in rmTBI patients. For CCI (model 6.3, American Instruments, Richmond, VA, United States), the extension of its impounder tip (diameter = 3 mm) was performed to the full impact distance, which was later located in the center of the disc surface and was reset to produce the impact. Subsequently, we charged the impact head at 5.0 m/s and the impact depth was 2.5 mm. After the impact, each mouse was put in the cage at 37 °C under good ventilation until recovery from consciousness. We performed a total of four impacts at 48-h intervals to establish a repetitive mild injury model. The sham mice underwent the same operating procedures except for the impact of all four times. During and after modeling, mice with impaired movement or difficulty eating were removed from the study, as were those with skull fractures or parenchymal injury (such as visible cerebral hemorrhage) found during brain harvesting.

### Experimental groups and treatment

**Fig. 1:**
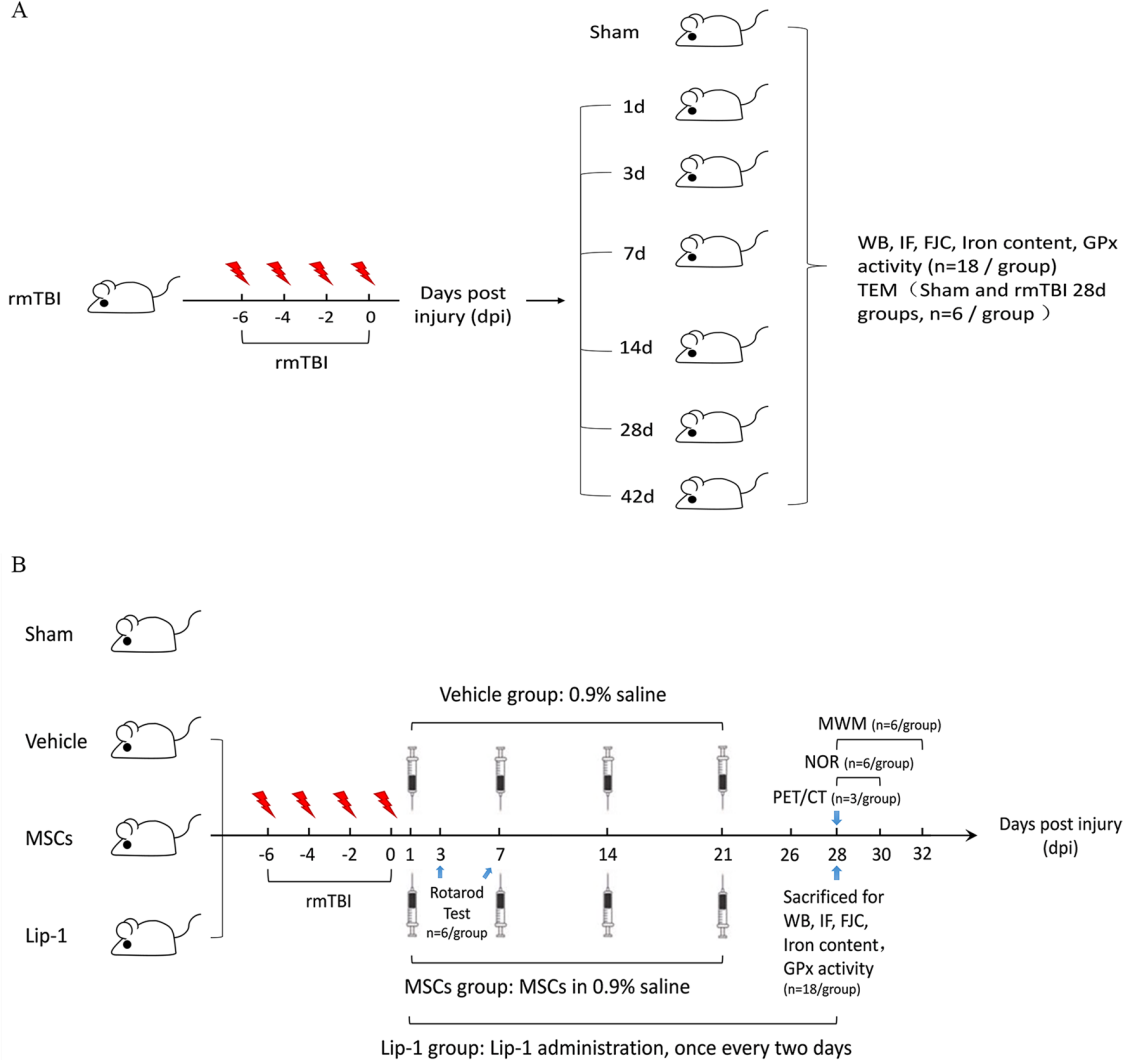
Schematic diagram of the study experimental design. Chart illustrating our study design including experimental groups, CCI, drug administration, WB, IF, FJC, iron content, GPx activity, TEM, PET/CT, rotarod test, MWM, NOR

To investigate time-dependent iron accumulation in cells, protein levels associated with iron metabolism, GPx activity, mitochondrial morphology, and neurodegeneration after rmTBI, we randomized mice in sham and other 6 groups according to different time points after rmTBI (1, 3, 7, 14, 28, 42 days post-injury). We further performed a Western blot (WB) assay, iron assay, GPx activity assay, transmission electron microscopy (TEM), fluoro-jade C (FJC) staining, and immunofluorescence (IF) assay (Fig. 1A).

To determine how MSCs affected iron metabolic disturbance, GPx inactivation, nerve cell injury, behavioral deficits, and protein levels after rmTBI, we classified the mice into 4 groups, including Sham, rmTBI + Vehicle (Vehicle), rmTBI + MSCs (MSCs), and rmTBI + Lip-1 (Lip-1, positive control). Vehicle mice were administered 0.9% saline (100 µL) via the tail vein, while MSCs mice were injected with 100 µL of suspension containing 1 × 10^6^ MSCs through the tail vein 24 h after rmTBI and once a week until killing [30, 31]. Lip-1 (S7699, Selleckchem), a specific and strong ferroptosis inhibitor, was diluted by 2% DMSO, 40% PEG 300, 2% Tween 80, and ddH_2_O. Mice in the Lip-1 group (10 mg/kg) were injected intraperitoneally 24 h after rmTBI and once every two days before killing [32]. Mice were subjected to the rotarod test on days 3 and 7 post-rmTBI. On day 28 post-rmTBI, some mice underwent a positron emission tomography/computed tomography (PET/CT) and behavioral tests, including the novel object recognition test (NOR) and Morris water maze (MWM) test, while others were killed for an iron assay, IF, FJC staining, GPx activity assay, and WB assay (Fig. 1B).

### MSC preparation

We obtained human umbilical cord blood-derived (hUCB) MSCs (CP-H165, Procell), enzymes (PB180229), and corresponding growth-supporting medium (CM-H165) from Procell Life Science & Technology Co. Ltd. Cell preparation and subculture were carried out following the specific instructions as reported [30]. The main steps are as follows: in the biosafety cabinet or super-clean platform, remove the original culture medium, add about 2 ml of PBS, gently shake the culture bottle to rinse the cells, remove the PBS, and discard. Add about 1 ml of trypsin, gently shake the culture bottle to infiltrate all cells, and keep it in the incubator for digestion for about 30 s. See that the cells are obviously separated and round under the microscope, then add 3 ml of hMSC culture medium to terminate digestion, blow the cells off and blow the cells in the liquid repeatedly to make them as single cell suspension as possible, collect cell suspension and centrifuge at 1000 rpm for 3 min, suck the supernatant and discard, add 10 ml of hMSC culture medium to resuspend the cells. Inoculate in a new culture flask, replenish the medium and use a breathable bottle cap for culture. Culture in a 37℃ constant temperature cell incubator with 5% CO2. The cell status was observed 24 h later and the fresh medium was replaced, and then the culture medium was replaced every 2–3 days. We used cells in the second and third passages for injection.

### NOR test

We performed the NOR test to evaluate the recognition memory of mice on days 28–30 post-rmTBI as reported [33]. The NOR tests are performed every morning in a quiet behavioral testing room (temperature 25 °C, humidity 56%). The experimental platform was an open field box (40 cm × 40 cm × 50 cm) (CleverSys, Reston, VA, USA), and we measured the time required for object recognition by each mouse within 10 min. This experiment included three steps: day 1, habituation phase: mice could study territory with freedom for 5 min with no object; day 2, recognition phase: two objects of the same shape, size, and color were placed on the left and right ends of a side wall, about 10 cm away from the two sides of the wall. Place the mice in the box with their backs to the two objects, each mouse explored to became familiar with these two objects in 10 min; day 3, test phase: a new object with different colors and shapes was used to replace one object, then the mice explored them for 10 min. An Anymaze video tracking system (Stoelting) equipped with a computer-connected digital camera was used to track the whole course of animal activities in training and experimental sessions of the NOR tests. We video-recorded all animal tests and determined the time spent exploring every object and the total time spent exploring the two objects. Then, we calculated the NOR index (NORI) to evaluate alterations in the recognition memory of mice in different groups. NORI = *t* (new)/*t* (old + new)*100%, where 't' represents the time spent exploring each object. Statistical analysis was also performed.

### Rotarod test

The rotarod test was performed on days 3 and 7 post-rmTBI as reported [34]. The test is performed every morning in a quiet behavioral testing room (temperature 25 °C, humidity 56%). Mice were trained for 2 days prior to the rotarod test. Each mouse was trained on a rotating rod machine (YLS-4D, Yiyan Technology Development, Jinan, China) at a speed of 4–40 rpm for at least 200 s, three times a day. The test was conducted on the third day, the maintenance of each mouse on the rod at 40 rpm for 10 min, and each mouse was given three tests, the average latency to fall from the rotating rod (rotarod latency) was recorded, and the mice unable to grasp the rod were given a latency of 0 s.

### MWM test

The MWM test was performed on days 28–32 post-rmTBI to determine changes in spatial memory and learning in four groups of mice (sham, vehicle, MSCs, Lip-1) as reported [29]. Consequently, each mouse was trained to find a hidden platform in opaque water. During the initial 4 days (training phase), we placed the platform (diameter = 10 cm) 1 cm below the water surface. Each mouse received 4 trials (90 s each) every day. Each mouse started in different quadrants in different trials under an identical starting pattern. This test measured the escape latency or time needed to find the hidden platform, which is the memory and spatial learning index (maximum 90 s). The mouse that was unable to find the platform within the initial 90 s was gently guided to the platform. Once a mouse was on the platform, it was allowed to sit on it for 15 s. On the fifth day, we performed the spatial probe test (SPT). We removed the platform and each mouse swam for 90 s. Thereafter, the video tracking system (Stoelting) was used to determine the frequency of platform crossing, the first latency to arrive at the platform location, and swimming speed.

### Glucose metabolism determined by ^18^F-fluoro-2-deoxy-D-glucose (FDG) and TAU metabolism determined by ^18^F-S16-TAU using PET/CT

After anesthesia administration, each mouse was given an intraperitoneal injection of ^18^F-fluoro-2-deoxy-d-glucose ([^18^F] FDG) or a tail vein injection of [^18^F] S16-TAU at 5 MBq. After 30 min, 2% isoflurane gas was applied within the oxygen flow to the mouse anesthesia. The mice were then placed on a micro-PET/CT scanner bed (Novel Medical, Beijing, China). The parameters for PET scan were as follows: matrix = 140 × 140; field of view (FOV) = 70 mm; and reconstruction agreement = PET-OSEM-Recon for 40 iterations. The parameters for CT examination were the following: tube current = 0.5 mA; tube voltage = 80 V; layer thickness = 0.18 mm; and FOV: 70 mm.

### Preparation of specimens

To perform IF staining, each mouse received 4% paraformaldehyde (PFA) transcardial perfusion and cold phosphate-buffered saline (PBS) for killing. Subsequently, we collected brain samples and kept them on ice, followed by 24-h PFA fixation, then dehydration with 15% sucrose for 24 h, and finally dehydration with 30% sucrose for 24 h. Each brain was then subjected to ice embedding within an optimal cutting temperature (Sakura, Torrance, CA, USA). Brain samples were cut into 8 µm and 12 µm coronal sections using a − 20 °C frozen slicer for FJC staining and IF staining, respectively.

For Western blotting, evaluation of iron levels, and determination of GPx activity, we killed the mice using cold PBS transcardial perfusion. We then harvested the brain tissues on ice, removed the microvessels, and preserved them using liquid nitrogen until measure.

### TEM

We used 2% PFA and 2% glutaraldehyde in 0.1 M sodium cacodylate to perfuse specimens collected from rmTBI 28 d group and sham group, followed by fixation with 2% osmium tetroxide and 1.6% potassium ferrocyanide in 0.1 M sodium cacodylate. The samples were cut to 1 mm^3^ thickness and stained with 2% uranyl acetate, followed by dehydration with ethanol along with the embedding of Eponate. Each section was placed on copper slot grids, followed by staining with lead citrate and uranyl acetate (2%). Images were acquired using a transmission electron microscope (7600; Hitachi, Tokyo, Japan).

### IF staining

After 30 min of fixation with 4% PFA at room temperature, each section was rinsed twice with PBS (5 min each). The section was then treated with 3% BSA for 30 min at room temperature to block nonspecific binding sites. Sections were incubated overnight at 4 °C with the following primary antibodies: GPx4 (1:1000; ab125066; Abcam) and MAB1281 (1:200; Sigma-Aldrich). The next day, the sections were rinsed with PBS, followed by another 1 h of incubation with secondary antibodies at room temperature. The nuclei were then stained with 4',6-diamidino-2-phenylindole (DAPI) (ab104139; Abcam). A fluorescence microscope (Olympus, Heidelberg, Germany) was used for viewing the stained sections and acquiring images. GPx4-positive cells were in three separate slides from each brain, with each slide containing three 200 × magnification fields from cerebral cortex and hippocampus.

### FJC staining

For detecting nerve cell degeneration, we used the FJC Ready-to-Dilute Staining Kit (FJC, TR-100-FJ, Biosensis, CA) for FJC staining according to specific protocols [35]. Briefly, brain sections were sequentially immersed with 1% NaOH, 70% ethanol, distilled water, and 0.06% potassium permanganate. After being washed with distilled water, the sections were stained with 0.0001% FJC working solution and DAPI. The sections were then rinsed and dried in the dark. Finally, they were cleared in xylene and slipped on the slices. Stained slices were observed and images were acquired under a fluorescence microscope (Olympus, Heidelberg, Germany). The number of FJC-positive cells was counted under a microscopic field of 400 × magnification in three visual fields that were randomly selected from three discrete sections of each sample.

### Evaluation of iron levels

The Iron Assay Kit (ab83366; Abcam) was used to assess iron levels. The detection process was performed according to the manufacturer’s protocol. The main steps are as follows: harvest tissue and wash tissue in cold PBS, then homogenize tissue in 4–10 volumes of Iron Assay Buffer using a Dounce homogenizer sitting on ice. Centrifuge at 16,000×*g* for 10 min. Collect the supernatant and keep on ice. Set up reaction wells: standard wells = 100 µL standard dilutions. Sample wells = 2–50 µL samples (adjust volume to 100 µL/well with Iron Assay Buffer). Add 5 µL Iron Reducer to each standard well, add 5 µL of Assay buffer to each sample. Mix and incubate standards and samples at 37 °C for 30 min. Add 100 µL Iron Probe to each well containing the iron standard and test samples. Mix and incubate at 37 °C for 60 min protected from light. Measure the output immediately on a colorimetric microplate reader (OD 593 nm).

### Evaluation of GPx activity

GPx activity was determined using the Glutathione Peroxidase Assay Kit (ab102530; Abcam). The detection process was performed according to the manufacturer’s protocol. The main steps are as follows: harvest tissue samples and wash tissue with cold PBS. Resuspend samples in 200 µL of cold Assay Buffer. Homogenize the samples quickly and centrifuge for 15 min at 4 °C at 10,000*g* using a cold microcentrifuge. Collect the supernatant and keep on ice. Set up reaction wells: Standard wells = 100 µL standard dilutions. Sample wells = 2–50 µL samples (adjust volume to 50 µL/well with Assay Buffer). Reagent control wells = 50 µL Assay Buffer. Immediately prior to use, prepare reaction mix for each reaction and mix enough reagents for the number of to be performed. Prepare a master mix of the reaction mix to ensure consistency. Add 40 µL of the reaction mixture to the sample and reagent control wells. Mix well and incubate at room temperature for 15 min. Add 10 µL cumene hydroperoxide solution, to the sample and reagent control wells. Mix well. Measure the output (A1) on a microplate reader at OD340 nm at T1. Incubate at 25ºC for 5 min. Protect against light. Measure the output (A2) on a microplate reader at OD340 nm at T2.

### Western blotting

We performed sodium dodecyl sulfate-polyacrylamide gel electrophoresis (SDS-PAGE) along with immunoblotting according to a previous description [27]. Amyloid precursor protein (APP; 1:1000; ab12269; Abcam), phospho-tau (p-Tau; 1:1000; 11,834; Cell Signaling Technology), Fpn (1:1000; NBP1–21502SS; NOVUS), tau-5 (1:1000; ab80579; Abcam), transferrin receptor (1:1000; ab214039; Abcam), and 4-hydroxynonenal (1:1000; ab46545; Abcam) were detected using 10% SDS-acrylamide gel. Meanwhile, NeuN (1:1000; ab177487; Abcam), glutathione peroxidase 4 (1:1000; ab125066; Abcam) and beta-amyloid 1–42 (Aβ1–42; 1:1000; ab201061; Abcam) were detected using 12% SDS-acrylamide gel. β-Actin (1:1000; ab8226; Abcam) and glyceraldehyde 3-phosphate dehydrogenase (GAPDH) (1:2000; 2118; Cell Signaling Technology) were used as endogenous references. The details of the antibodies are shown in Table 1. We used the ChemiDoc XRS + Imaging System (Bio-Rad, Hercules, CA, USA) for the densitometric analysis. Image J software was used to measure the band area.

**Table 1:** Details of the reagents used

Reagents	Brand	Catalogue number
Anti-APP Antibody	Abcam	ab12269
Phospho-TAU Antibody	Cell Signaling Technology	11834
Ferroportin Antibody	NOVUS	NBP1–21502SS
Anti-TAU Antibody	Abcam	ab80579
Anti-Transferrin Receptor Antibody	Abcam	ab214039
Anti-4 Hydroxynonenal Antibody	Abcam	ab46545
Anti-NeuN Antibody	Abcam	ab177487
Anti-Glutathione Peroxidase 4 Antibody	Abcam	ab125066
Anti-beta Amyloid 1–42 Antibody	Abcam	ab201061
Anti-beta Actin Antibody	Abcam	ab8226
GAPDH	Cell Signaling Technology	2118
DAPI	Abcam	ab104139
Liproxstatin-1	Selleckchem	S7699
FJC Ready-to-Dilute Staining Kit	Biosensis	TR-100-FJ
Iron Assay Kit	Abcam	ab83366
Glutathione Peroxidase Assay Kit	Abcam	ab102530

### Statistics analysis

Data are represented as mean ± standard deviation (SD). GraphPad Prism 9.0 (GraphPad Software) and SPSS 26.0 were used for plotting and statistical analysis. Except for escape latency, all data were analyzed using one-way analysis of variance (ANOVA) followed by Dunnett’s post hoc test, Tukey’s post hoc test, Dunnett’s T3 post hoc test, and LSD post hoc test. Escape latency data were compared using repeated-measures ANOVA and multicomparison. Spearman’s correlation analyses were used to correlate GPx activity and FJC-positive cells. A *P* value < 0.05 was considered statistically significant.

## Results

### The occurrence of ferroptosis was detected after rmTBI

**Fig. 2:**
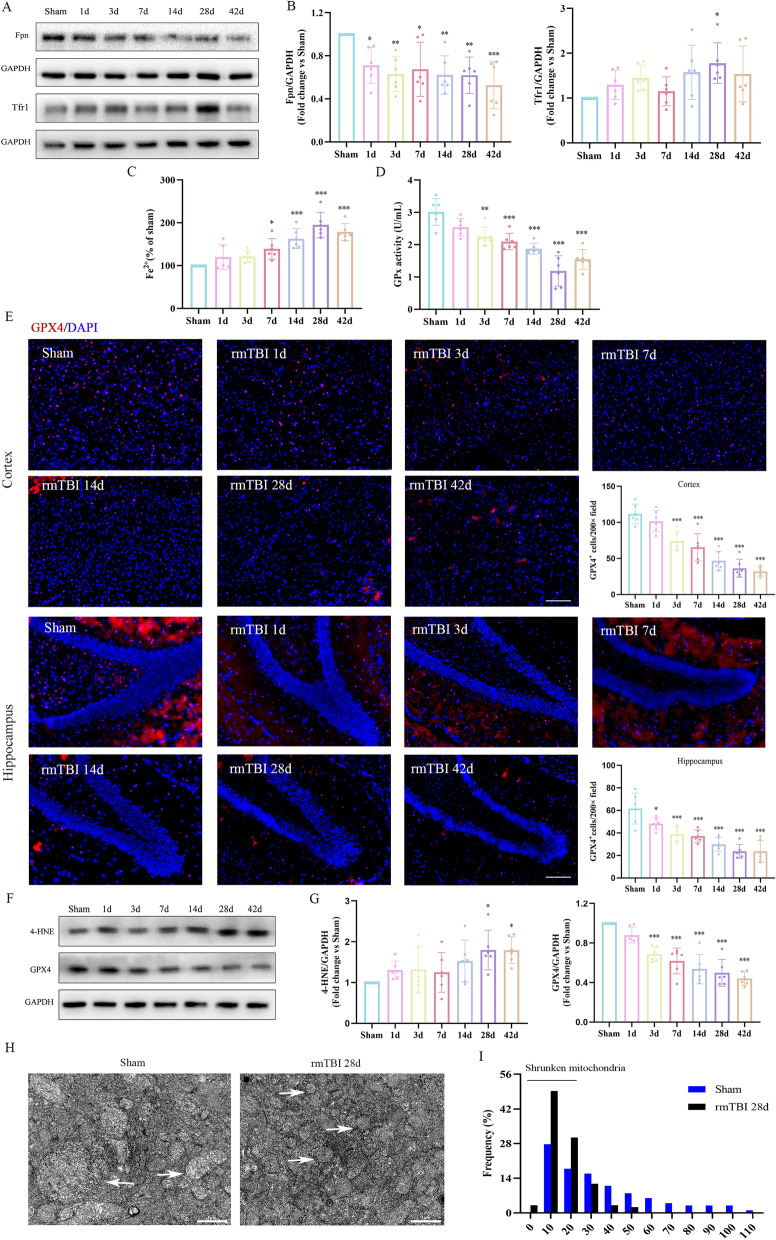
The occurrence of ferroptosis was detected after rmTBI. **A**,** B** Western blot was performed to observe the time course of changes in the expression of iron metabolism-related proteins (Tfr1 and Fpn). Protein expression was normalized to GAPDH and expressed as fold change of sham. **C**,** D** The time course of changes in Fe^2+^ content and GPx activity. **E** Representative immunofluorescence staining of GPx4-positive cells in the cortex and hippocampus at different time points after rmTBI and quantitative analysis of GPx4-positive cells. Red indicates positive GPx4 staining, and blue indicates positive DAPI nuclear staining. Scale bars = 100 μm. **F**,** G** Western blot was performed to observe the time course of changes in the expression of 4-HNE and GPx4. Protein expression was normalized to GAPDH and expressed as fold change of sham. **H** The ultrastructure of the cortex on day 28 after rmTBI in mice was captured by transmission electron microscopy. The white arrow indicates mitochondria. **I** Histogram showing the frequency of mitochondrial area in the cortex. Number of mitochondria, sham: *n* = 101; rmTBI 28 d: *n* = 128. Scale bars = 1.0 μm. Data are expressed as mean ± SD (*n* = 6) and analyzed using one-way ANOVA with Dunnett’s post hoc test (**B**-**E**, **G**). *P < 0.05, **P < 0.01, and ***P < 0.001 versus sham

Aberrant iron homeostasis is associated with ferroptosis, which induces the pathology of the CNS. To determine whether abnormal iron homeostasis occurs after rmTBI, western blotting was performed and non-heme iron levels were evaluated. We first evaluated the levels of iron metabolism-associated proteins including Tfr1 and Fpn in rmTBI groups of 1, 3, 7, 14, 28, 42 days post-injury and the sham group. As shown in Fig. 2A, B, the Tfr1 level increased significantly on day 28 post-rmTBI compared with those in the sham group. The difference was not significant between the sham and other time points groups. On the contrary, the levels of Fpn decreased significantly after day 1 post-rmTBI until day 42 post-rmTBI compared with those in the sham group. We then evaluated the Fe^2+^ levels, which indicated iron overload and produced hydroxyl radicals with high reactivity by the Fenton reaction [36]. We found that Fe^2+^ levels in rmTBI mice were significantly higher than those of mice in the sham group after day 7 post-rmTBI, and peaked on day 28 post-rmTBI (Fig. 2C). Together, abnormal Tfr1 and Fpn levels led to an imbalance in iron transport and promoted iron accumulation after rmTBI. These results suggested that iron metabolism disorder occurs after rmTBI.

A deficiency in GPx activity is assumed to be one of the causes of ferroptosis in cells [18, 36]. We detected GPx activity at different time points after rmTBI and found that GPx activity decreased significantly on day 3 post-rmTBI compared with the sham group. The lowest GPx activity was detected on day 28 post-rmTBI (Fig. 2D). IF staining was used to evaluate the number of GPx4-positive cells. We found that the number of GPx4-positive cells decreased significantly from day 1 to day 42 post-rmTBI in the hippocampus and decreased significantly from day 3 to day 42 post-rmTBI in cortex (Fig. 2E). Similarly, GPx4 protein levels decreased significantly from day 3 to day 42 post-rmTBI after injury compared with those of the sham group (Fig. 2F, G). These results indicate that both activity and the quantity of GPx4 decreased significantly after rmTBI.

A reactive product derived from lipid peroxidation, 4-HNE, can indicate the occurrence of ferroptosis. The levels of 4-HNE protein increased considerably on days 28 and 42 post-rmTBI compared with those of the sham group (Fig. 2F, G). TEM was used to determine ferroptosis-related morphology. Mitochondrial shrinkage and mitochondrial cristae decreased or disappeared on day 28 post-rmTBI compared with those of the sham group (Fig. 2H, I).

To summarize, we detected characteristic changes in ferroptosis after rmTBI, such as abnormal iron metabolism, GPx inactivation, decreased expression of GPx4, increased lipid peroxidation products, and mitochondrial shrinkage. These results suggest the occurrence of ferroptosis after rmTBI.

### Neuronal loss and neurodegeneration increased after rmTBI

**Fig. 3:**
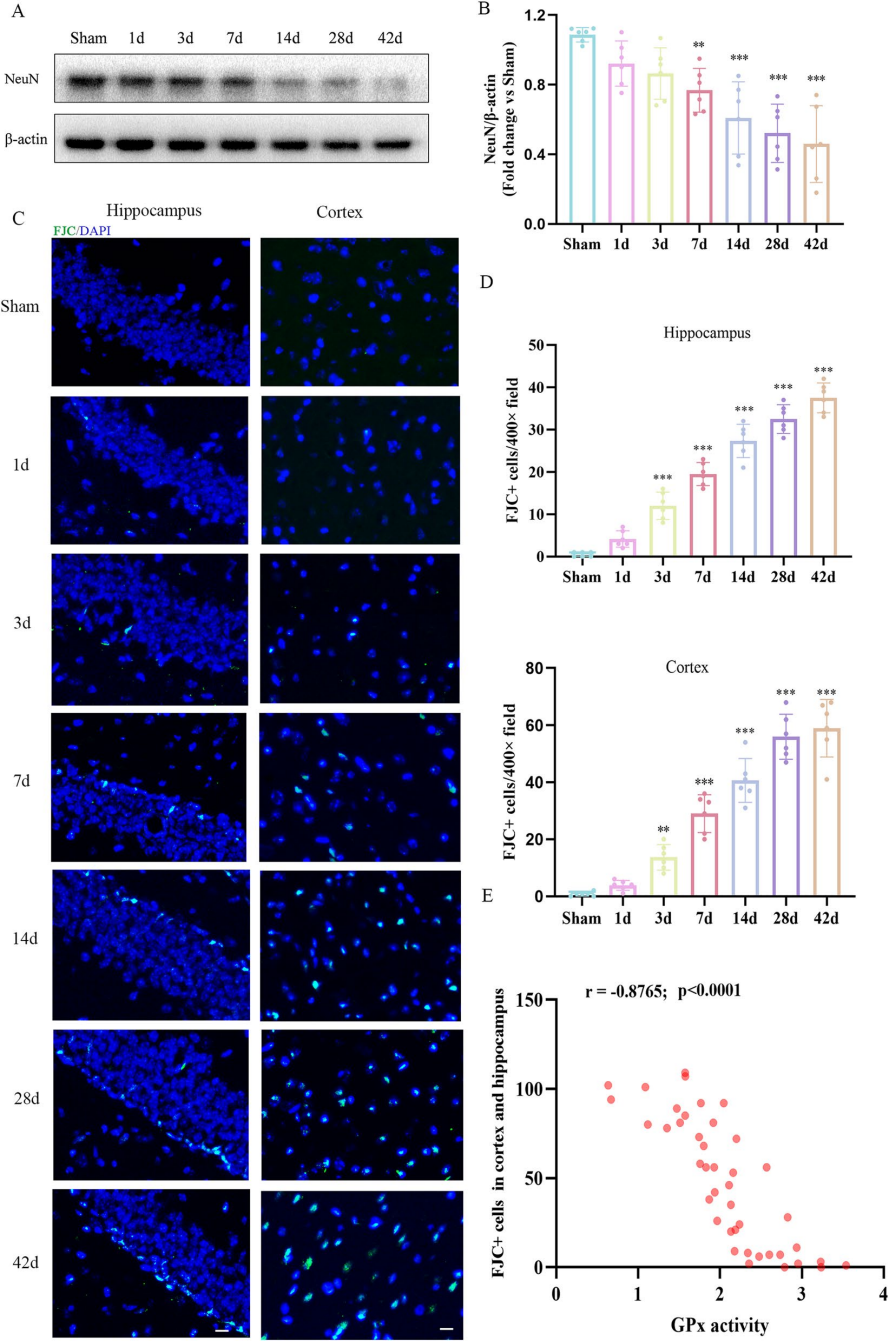
Neuronal loss and neurodegeneration increased after rmTBI. **A**,** B** Western blot was performed to observe the time course of changes in the expression of NeuN. Protein expression was normalized to β-actin and expressed as a fold change of sham. **C** Representative immunofluorescence staining of FJC-positive cells in the cortex and hippocampus at different time points after rmTBI. Green indicates FJC-positive staining, and blue indicates positive DAPI nuclear staining. Scale bar = 25 μm. **D** Quantitative analysis of FJC-positive cells. Data are expressed as mean ± SD (*n* = 6) and analyzed using one-way ANOVA followed by Dunnett’s post hoc test (B, D). **P < 0.01, and ***P < 0.001 versus sham. **E** The correlations between FJC-positive cells in the cortex and hippocampus and GPx activity were evaluated using Spearman’s correlation analysis (*r* = − 0.8765, *P* < 0.0001)

To determine neuronal loss and neurodegeneration after rmTBI, we performed a western blot assay and FJC (degenerating neuron marker) staining. We found that the NeuN protein levels were significantly lower than that of the sham group from day 7 post-rmTBI to day 42 post-rmTBI (Fig. 3A, B). This result suggested a significant increase in neuronal loss after rmTBI.

We performed FJC staining to evaluate nerve cell degeneration within the hippocampus and cortex. According to the results of the FJC staining, compared with the sham group, the number of FJC-positive cells increased significantly within both the cortex and the hippocampus from day 3 post-rmTBI to day 42 post-rmTBI (Fig. 3C, D). Therefore, we hypothesized that significant neuronal loss and neurodegeneration occurred after rmTBI associated with persistent cognitive impairment.

Linear correlation analysis showed that GPx activity was negatively correlated with the number of FJC-positive cells, suggesting that ferroptosis was may be associated with neurodegeneration (Fig. 3E).

### MSC treatment attenuates persistent cognitive deficits induced by rmTBI in mice

**Fig. 4:**
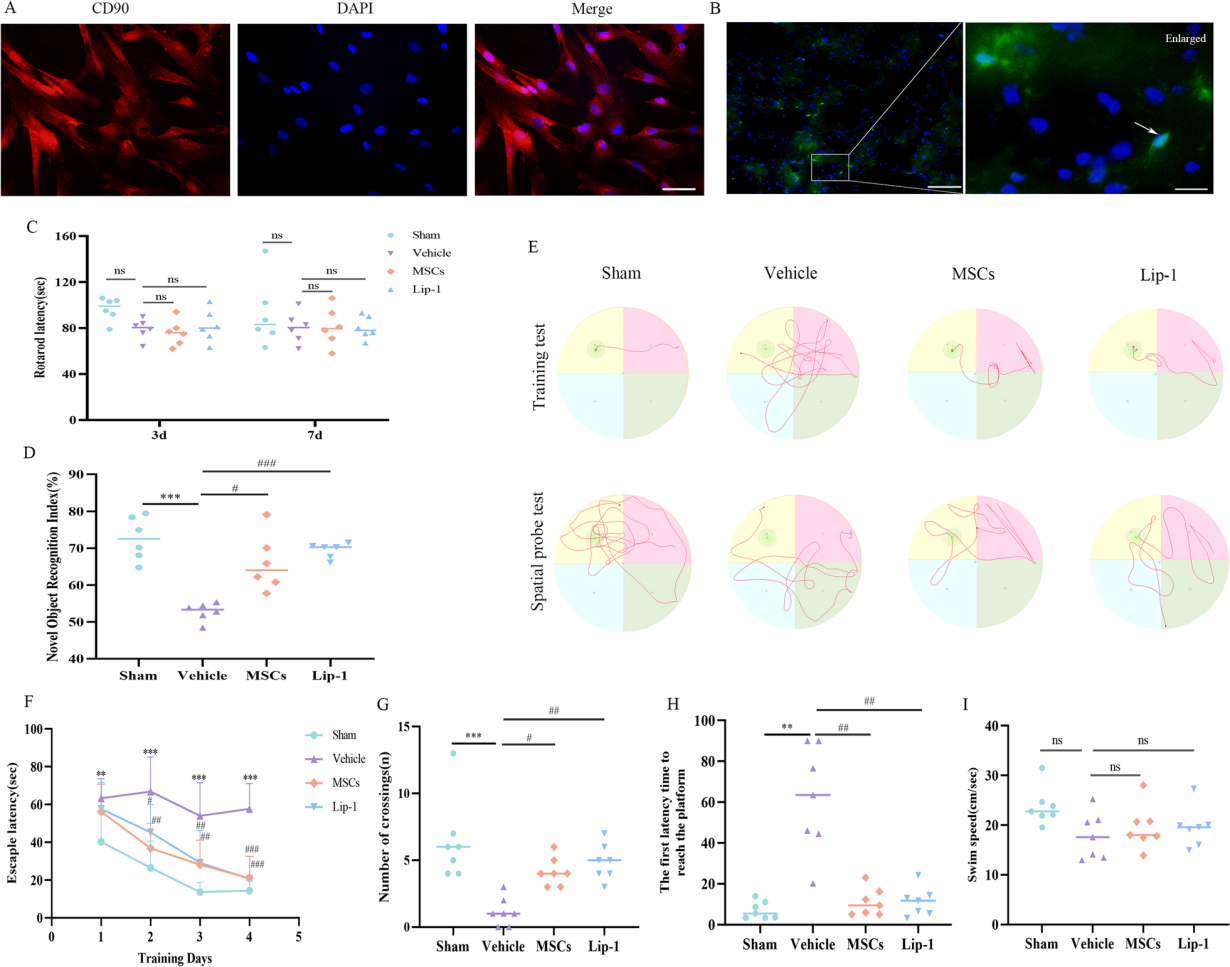
MSC treatment attenuates persistent cognitive deficits induced by rmTBI in mice. **A** Representative immunofluorescent images of CD90 + cells were used to identify MSCs. Scale bar = 50 µm. **B** Representative immunofluorescent images of MAB1281 + cells were used to observe the migration of MSCs. Scale bar (left) = 100 µm; Scale bar (right) = 15 µm. **C** The rotarod test was tested on days 3 and 7 post-rmTBI. **D** The NOR test was tested on days 28–30 post-rmTBI. **E**–**I** The MWM test was performed on days 28–32 post-rmTBI. Representative training and spatial probe traces of mice in the sham, vehicle, MSCs, and Lip-1 groups (**E**). Escape latencies during the training period of MWM (**F**). The frequency of crossing the hidden platform during the probe trial of MWM (**G**). The first latency time to reach the platform during the probe trial of MWM (H). Swim speed during the probe trial of MWM (**I**). Data are expressed as mean ± SD and analyzed using one-way ANOVA with Dunnett’s T3 post hoc test and Tukey’s post hoc test (**C**, **D**, **G**, **H**, **I**). Repeated measures ANOVA with multiple comparisons was used for comparisons of escape latency (**F**). For vehicle group: **P < 0.01, ***P < 0.001 vs sham group; for MSCs group: ^#^*P* < 0.05, ^##^*P* < 0.01, ^###^*P* < 0.001 vs vehicle group; for Lip-1 group: ^##^*P* < 0.01, ^###^*P* < 0.001 vs vehicle group

IF staining detected the surface-specific marker CD90 of MSCs (Fig. 4A). MAB1281(human nuclear antibody) can be used to identify human cells in xenotransplantation models, because it reacts specifically with human cells and does not respond to mouse cells. MAB1281 staining was performed to measure exogenous MSCs within the mice. MAB1281 + cells were found in the brains of mice treated with MSC (Fig. 4B). This result showed that MSCs can penetrate the blood–brain barrier (BBB) after rmTBI, which is consistent with a previous study [37] and may be related to increased BBB permeability after injury.

The rotarod task was performed on days 3 and 7 after rmTBI to assess mouse motor coordination. The performance of the rotarod did not show significance difference among the four groups, suggesting that rmTBI mice did not have impaired motor coordination (Fig. 4C).

To determine the effect of MSCs on rmTBI-induced cognitive impairment, NOR and MWM tests were performed to evaluate mouse memory and learning ability. The NOR test was conducted on days 28–30 after rmTBI, the effect of MSCs on rmTBI mouse recognition memory was evaluated by calculating the NORI. The NORI value of the vehicle group decreased markedly compared with the sham group, indicating impaired memory. Compared with the vehicle group, the MSCs and Lip-1 groups had a long time for novel object recognition and had significantly higher NORI. This indicated that impaired cognitive faculties in rmTBI mice were attenuated by MSC or Lip-1 treatment (Fig. 4D).

The MWM test was conducted on days 28–32 after rmTBI, vehicle mice exhibited an impaired spatial learning and memory capacity compared with sham mice, and treatment with MSC or Lip-1 significantly improved spatial learning in rmTBI mice, as evidenced by their swimming path and their learning curve (Fig. 4E, F). The results revealed that during the training phase, sham mice showed a training-related reduction in escape latency, while vehicle mice showed significantly longer escape latency compared with sham mice, and the escape latency of MSC- or Lip-1-treated mice was significantly shorter compared with vehicle counterparts (Fig. 4F). Furthermore, in the probe test, compared with the sham group, the vehicle group showed a significant decrease in platform-crossing frequencies (Fig. 4G) and a marked increase in the first latency time to reach the platform (Fig. 4H), indicating impaired spatial learning and memory function. However, significantly increased platform-crossing frequencies (Fig. 4G) and markedly decreased the first latency time to reach the platform (Fig. 4H) were shown in the MSCs and Lip-1 groups compared with vehicle counterparts, indicating recovery of spatial learning and memory functions. Furthermore, we compared the average swim speeds between the four groups of mice and found no significant differences (Fig. 4I).

Taken together, vehicle mice showed impaired spatial learning and memory function compared with sham mice, whereas MSC or Lip-1 treatment significantly attenuated persistent cognitive deficits induced by rmTBI in mice.

### MSC treatment reduced pathological protein deposition and promoted glucose metabolism after rmTBI

**Fig. 5:**
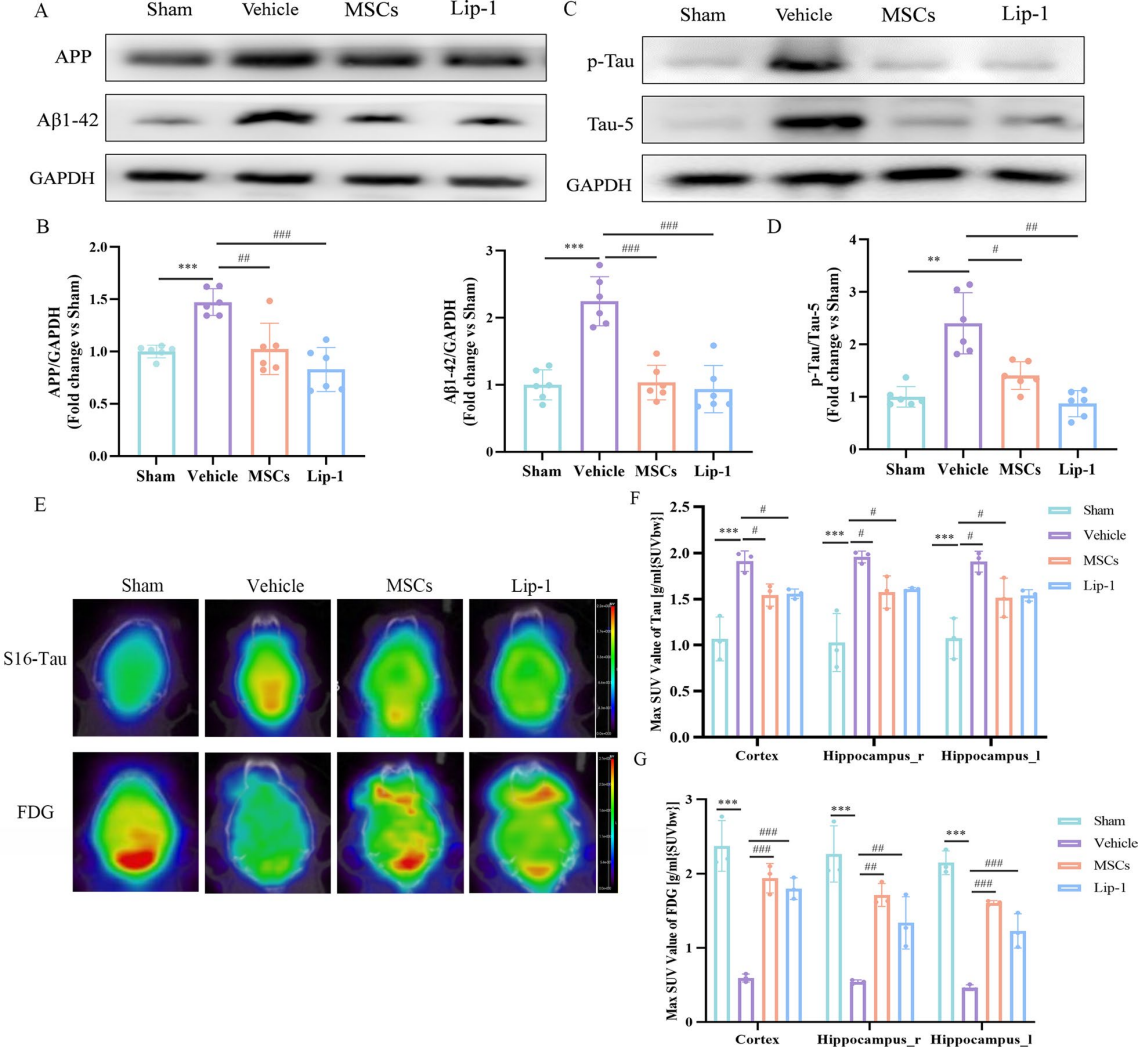
MSC treatment reduced pathological protein deposition and promoted glucose metabolism after rmTBI. **A**–**D** Western blot for APP, Aβ1-42, p-Tau, Tau-5 was performed on day 28 after rmTBI. Protein expression was normalized to GAPDH and expressed as a fold change in sham. Data are expressed as mean ± SD (*n* = 6) and analyzed using one-way ANOVA with Tukey’s post hoc test (**B**) and one-way ANOVA with Dunnett’s T3 post hoc test (**D**). **E**–**G** Reconstructed S16-TAU PET/CT images and [^18^F] FDG PET/CT images of mice in the sham, vehicle, MSCs and Lip-1 groups (**E**). Bar graph showing maximum SUV values in different regions of the brain of mice, including the cortex and hippocampi. Data are expressed as mean ± SD (*n* = 3) and analyzed using one-way ANOVA with the LSD post hoc test (**F**, **G**). For vehicle group: **P < 0.01, and ***P < 0.001 vs sham group; for MSCs group: ^#^*P* < 0.05, ^##^*P* < 0.01, and ^###^*P* < 0.001 vs vehicle group; for Lip-1 group: ^##^*P* < 0.01, ^###^*P* < 0.001 vs vehicle group

RmTBI is a critical risk factor for the occurrence and development of neurodegeneration, which is characterized by increased deposition of Aβ and tau proteins and cognitive impairment [38]. WB was used to verify changes in pathological proteins after rmTBI and the effect of MSCs on these changes. We observed that pathological protein levels, including APP, Aβ1–42, and p-Tau / Tau-5, were significantly elevated in the vehicle group compared with the sham group, while their levels decreased significantly within the MSCs and Lip-1 groups compared with the vehicle group (Fig. 5A–D).

Furthermore, we used small animal PET/CT to observe alterations in mouse cerebral imaging. Cerebral Tau expression was detected using the [^18^F] S16-tau probe and as a result, the standardized uptake value (SUV) values of S16-tau increased markedly within the cortices and hippocampi in the vehicle group compared with the sham group. However, these values were significantly lower in the MSCs and Lip-1 groups compared with the vehicle group (Fig. 5E, F), suggesting increased Tau protein deposition in the brain of rmTBI mice, which could be significantly reduced by MSC or Lip-1 treatment. Furthermore, from the [^18^F] FDG-SUV perspective, the vehicle group had significantly lower SUV values within the cortices and hippocampi compared with the sham group; these values were much higher in the MSCs and Lip-1 groups compared with the vehicle group (Fig. 5E, G), suggesting that the treatment with MSC or Lip-1 rescued the reduction induced by rmTBI in glucose metabolism.

All these results suggest that MSC treatment reduces intracerebral pathological protein levels while promoting glucose metabolism after rmTBI.

### MSCs treatment rescued rmTBI-induced neurodegeneration and neuronal loss

**Fig. 6:**
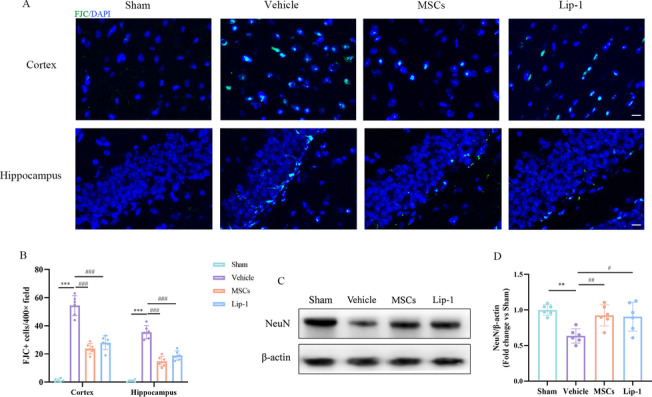
MSCs treatment rescued rmTBI-induced neurodegeneration and neuronal loss. **A** Representative immunofluorescence staining of FJC-positive cells in the cortex and hippocampus of sham, vehicle, MSCs, and Lip-1 groups. Green indicates FJC-positive staining, and blue indicates positive DAPI nuclear staining. Scale bar = 25 μm. **B** Quantitative analysis of FJC-positive cells. **C**,** D** Western blot analysis of NeuN proteins in the sham, vehicle, MSCs, Lip-1 groups. Protein expression was normalized to β-actin and expressed as a fold change of sham. Data are expressed as mean ± SD (*n* = 6) and analyzed using one-way ANOVA with Tukey’s post hoc test. For vehicle group: **P < 0.01, and ***P < 0.001 vs sham group; for MSCs group: ^##^*P* < 0.01, and ^###^*P* < 0.001 vs vehicle group; for Lip-1 group: ^#^*P* < 0.05, ^###^*P* < 0.001 vs vehicle group

To better study the effect of MSCs on rmTBI-mediated neuronal damage, FJC staining and WB were performed to detect neurodegeneration and neuronal survival. In terms of FJC, the number of FJC-positive cells decreased significantly after MSC or Lip-1 treatment, suggesting decreased neurodegeneration (Fig. 6A, B). The WB results showed that MSC and Lip-1 treatments could significantly rescued rmTBI-induced neuronal loss (Fig. 6C, D). This indicated that MSC treatment could promote neuronal survival and reduce neurodegeneration after rmTBI.

### MSC treatment inhibits rmTBI-induced ferroptosis

**Fig. 7:**
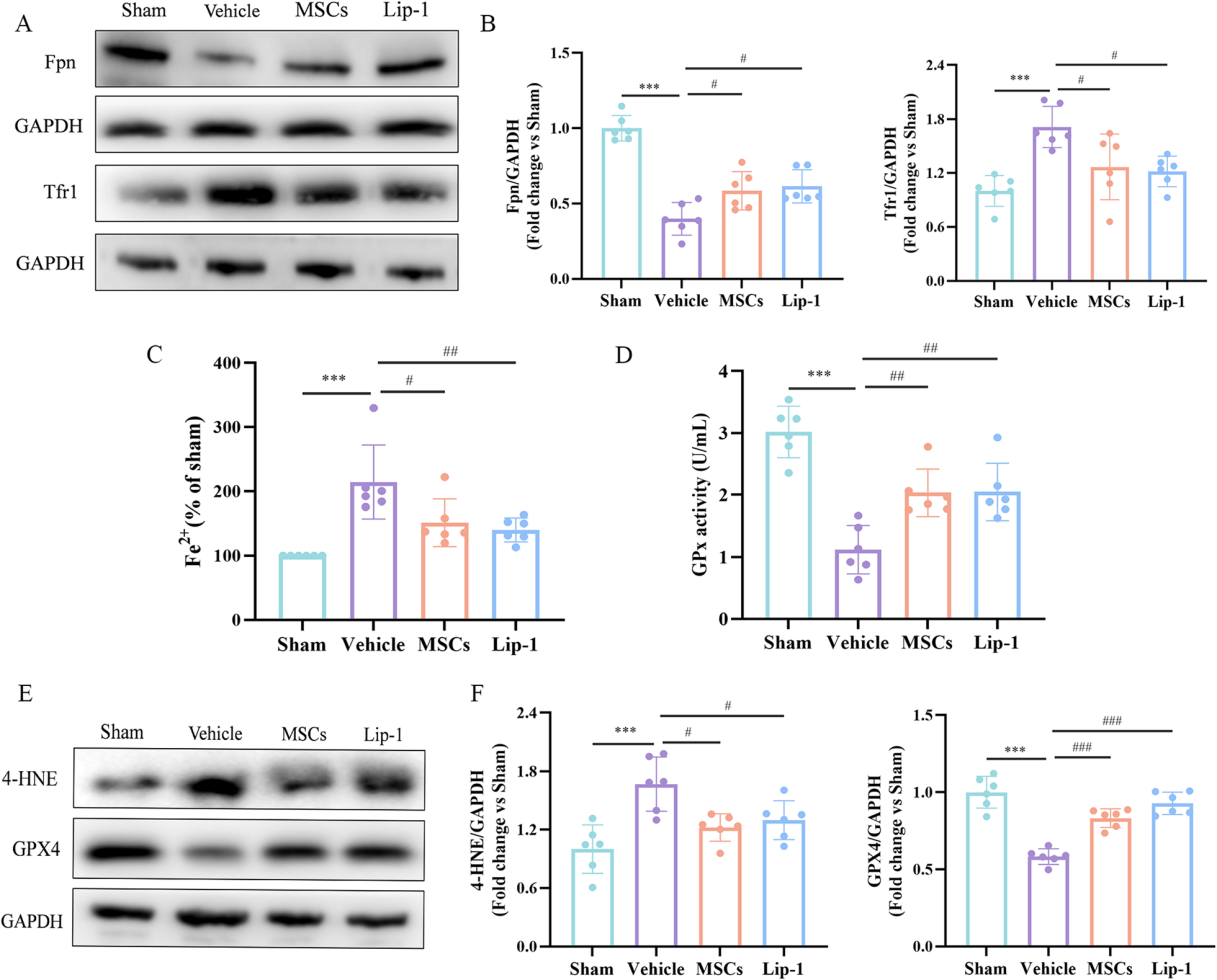
MSCs treatment inhibit rmTBI-induced ferroptosis. **A**,** B** Western blot analysis of the Fpn, Tfr1 proteins in sham, vehicle, MSCs, Lip-1 groups. Protein expression was normalized to GAPDH and expressed as fold change of sham. **C**,** D** The levels of Fe^2+^ content and GPx activity in the sham, vehicle, MSCs, Lip-1 groups. **E**,** F** Western blot analysis of the 4-HNE and GPx4 proteins in sham, vehicle, MSCs, Lip-1 groups. Protein expression was normalized to GAPDH and expressed as fold change of sham. Data are expressed as mean ± SD (*n* = 6) and analyzed using one-way ANOVA with Tukey’s post hoc test. For vehicle group: ***P < 0.001 vs sham group; for MSCs group: ^#^*P* < 0.05, ^##^*P* < 0.01, ^###^*P* < 0.001 vs vehicle group; for Lip-1 group: ^#^*P* < 0.05, ^##^*P* < 0.01, ^###^*P* < 0.001 vs vehicle group

To determine the role of MSCs in the rescue of rmTBI-mediated ferroptosis, iron levels, GPx activity assays, and WB were performed. As a result, MSC treatment remarkably suppressed rmTBI-induced up-regulation of Tfr1 and down-regulation of Fpn (Fig. 7A, B). We also found that MSC treatment significantly inhibited the up-regulation of Fe^2+^ content induced by rmTBI and markedly mitigated the decrease in GPx activity (Fig. 7C, D). Similarly to the result of the GPx activity assays, compared with the vehicle group, MSC treatment markedly increased GPx4 protein expression (Fig. 7E, F). Subsequently, MSC treatment significantly prevented the expression of 4-HNE proteins (Fig. 7E, F). Similar results were obtained in the Lip-1 group.

Taken together, MSC treatment potently regulates protein levels associated with iron metabolism, inhibits iron accumulation, restores GPx activity and GPx4 quantity, and prevents lipid peroxidation, thus rescuing rmTBI-induced ferroptosis. These suggest that ferroptosis inhibition may be an important mechanism for MSCs to reduce neurodegeneration and ameliorate persistent cognition impairment post-rmTBI.

## Discussion

To date, none of the neuroprotective therapies are effective for persistent impaired cognition among patients with rmTBI. Understanding the complicated mechanisms of rmTBI and chronic sequelae is of great importance, and it is urgent to find new effective therapeutic strategies. MSCs, pluripotent SCs, have been extensively investigated and are the preferred SCs for regenerative therapy. Here, we provide evidence that MSCs improve persistent cognitive function within the mouse model of rmTBI, partially by suppressing ferroptosis.

Several studies have evaluated how MSCs affect cellular, histopathological, and behavioral processes in many CNS diseases in the context of ROS markers, inflammatory factors, pathological proteins, and impaired cognition [39, 40]. MSCs have several protective activities in the management of CNS diseases through the production of exosomes and growth factors or the mitigation of neuroinflammation [23]; however, data on how MSCs affect cognitive function post-rmTBI are not available.

Ferroptosis has been discovered as a new form of cell death in recent years, which has an important effect on cancer occurrence and embryogenesis [10, 41]. Ferroptosis is present in various models of CNS disease, such as PD, AD, intracerebral hemorrhage, and post-traumatic epilepsy [13]. According to previous studies, ferroptosis participates in secondary injury after TBI, but its exact role in TBI and rmTBI and the effect of MSCs on resisting ferroptosis are unclear [18, 19]. According to this study, rmTBI caused alterations in ferroptosis-associated biomarker levels, and treatment with both MSC and Lip-1 (the ferroptosis inhibitor) markedly mitigated rmTBI-mediated ferroptosis, nerve cell injury, pathological protein deposition, and cognitive impairment.

This study used hUCB-MSC because they exhibit great benefits for cell storage, transplantation, and procurement compared with bone marrow-derived MSCs [42, 43]. Two recent studies have supported the approach to MSC administration in our study [30, 31]. Ferroptosis is inhibited by inhibitors of lipid ROS (Lip-1 and ferrostatin-1), lipophilic antioxidants (α-tocopherol, β-carotene, and butylated hydroxytoluene), as well as iron chelators (desferrioxamine) [18]. The researchers tested more than 40,000 drug-like small molecules in drug-inducible ferroptosis mice models and found a yet unrecognized class of spiroquinoxalinamine derivatives, including Lip-1. Compared with other ferroptosis inhibitors, Lip-1 can inhibit ferroptosis in the low nanomolar range and significantly improve cell viability without interfering with other classical types of cell death, showing significant effectiveness and specificity for ferroptosis [44].

Iron accumulation is a characteristic feature of ferroptosis. Iron is the fundamental element in nearly all organisms, as it participates in various metabolic processes [45]. Under physiological conditions, iron exists in nerve cells in the form of transferrin (Tf) as Fe^3+^, and the complex of the Tf–transferrin receptor (Tf–Tfr) functions in absorbing Fe^3+^ within the brain. The low pH value within the endosome disrupts the Tf-Fe^3+^–Tfr complex, which releases Fe^3+^, and subsequently Fe^3+^ will be reduced to Fe^2+^ and transferred to the cytoplasmic labile iron pool. On the other hand, Fpn modulates Fe^2+^ export and maintains the balance of iron metabolism together with the Tf–Tfr complex [13]. The imbalance of iron metabolism can lead to aberrant iron accumulation, which generates toxicity to brain-specific cells (including microglial cells, astrocytes, and neurons) [46]. The present study suggested that rmTBI resulted in a decrease in Fpn level and an increase in Tfr1 level and Fe^2+^ content. Interestingly, both MSCs and Lip-1 attenuated these aforementioned alterations. It is speculated that MSCs may reduce Fe^2+^ accumulation by regulating iron transport. However, the specific molecular mechanism by which MSCs prevent the up-regulation of the Tfr1 level and the down-regulation of the Fpn level after rmTBI is unclear and should be further investigated in future studies.

**Fig. 8:**
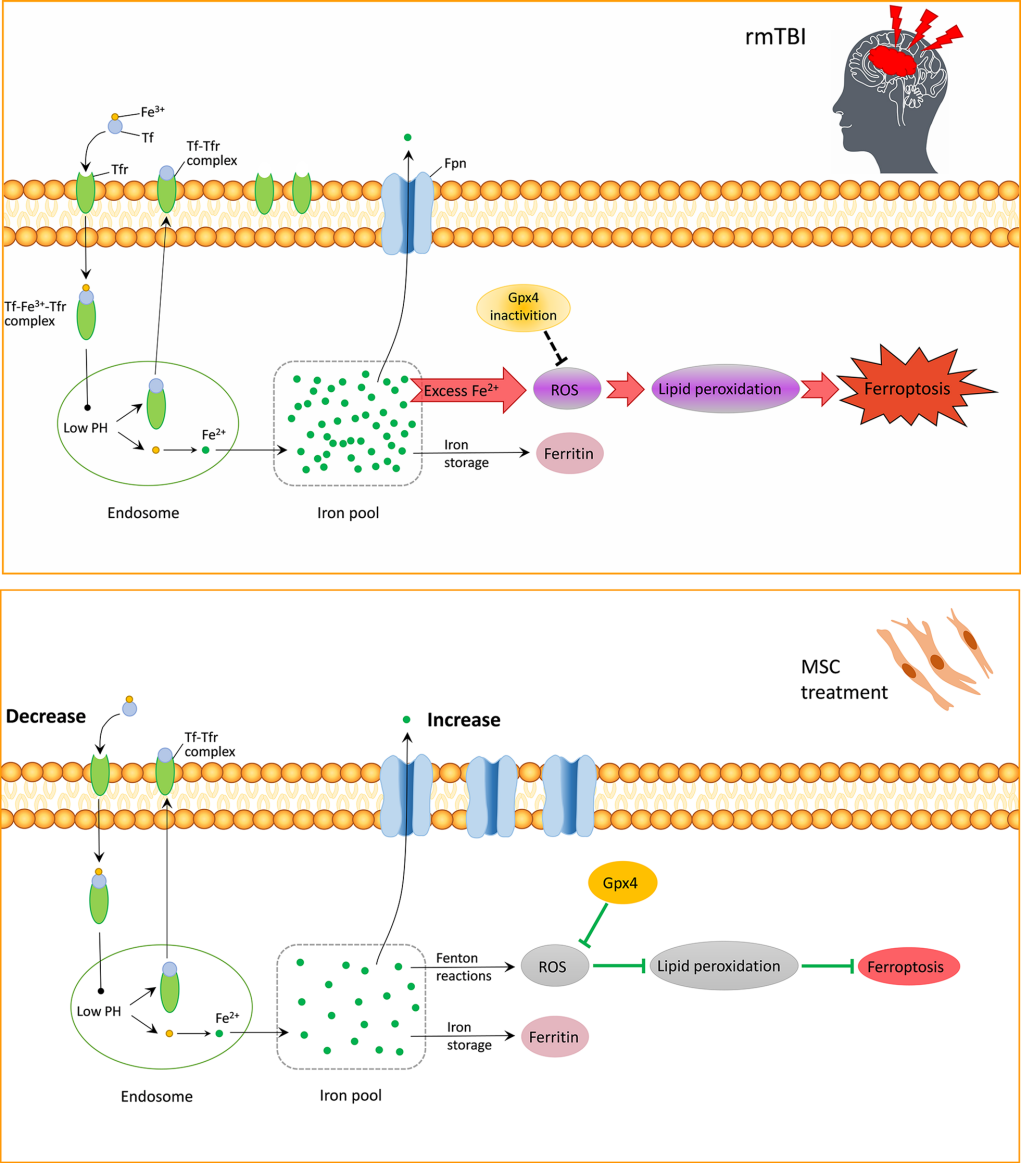
The schematic representation of MSC treatment inhibits rmTBI-induced ferroptosis

Lipid peroxidation is another characteristic of ferroptosis. Excessive Fe^2+^ generation after TBI breaks cell homeostasis and accelerates ROS generation through Fenton reactions, thus inducing cell injury. The brain shows a high vulnerability to excessive ROS production due to low antioxidants and high metabolism. Intracerebral oxidative injury is presented primarily as lipid peroxidation due to the increase in the content of polyunsaturated fatty acids within the membrane-abundant structure [47]. Lipid peroxides will degrade to toxic aldehydes such as 4-HNE and malonaldehyde, thus affecting cell membrane permeability and fluidity. Gpx4 inhibits lipid peroxidation by directly reducing hydroperoxides within membrane lipids and is considered a key regulator or target of ferroptosis [48]. Previous studies have shown that inhibiting GPx4 by gene knockout or drug inactivation caused ferroptosis [49]; however, whether GPx4 is dysfunctional after rmTBI has not been investigated. We found that 4-HNE protein expression increased on days 28 and 42 post-rmTBI, but less 4-HNE expressions were observed within the Lip-1 and MSCs groups compared with the vehicle group, indicating that MSCs could reduce lipid peroxidation. Furthermore, this study found that the expression of the GPx4 protein and GPx activity decreased significantly on day 3 post-rmTBI and continued to decrease gradually thereafter. MSCs and Lip-1 inhibited overexpression of 4-HNE, inactivation of GPx, and decrease in the protein levels of GPx4 induced by rmTBI. Through TEM, we observed distinct morphological changes characteristic of ferroptosis in the cerebral cortex of mice on day 28 post-rmTBI, manifested by mitochondrial shrinkage and disappearance of the mitochondrial bilayer membrane structure. In conclusion, iron accumulation, decrease in GPx4 protein levels and GPx activity, increase in lipid peroxidation products, and changes in mitochondrial ultrastructure provide potent evidence of ferroptosis induced by rmTBI. Furthermore, MSCs can inhibit ferroptosis by regulating iron metabolism, GPx activation, and lipid peroxidation (Fig. 8).

As suggested in several reports, ferroptosis occurs during cognitive impairment. The up-regulation of 4-HNE was observed within some regions of the brain in patients with slightly impaired cognition indicates lipid peroxidation [50]. On the other hand, iron accumulation is associated with impaired cognition among mTBI patients [51]. Furthermore, GPx4 depletion aggravated impaired cognition [52]. Many studies have indicated the protection of suppressing ferroptosis during cognitive impairment. For example, Fer-1 injected into the cerebral ventricle markedly decreased nerve cell degeneration while improving the long-time cognition in the mouse model of CCI [53]. Similarly, GPx4BIKO mice fed the vitamin E-deficient diet exhibited a faster rate of hippocampal neurodegeneration and cognitive impairment compared with their healthy counterparts; however, mice injected with ferroptosis inhibitor exhibited reduced neurodegeneration [52]. These findings indicated that ferroptosis was the neurodegeneration-deriving mechanism. Through the MWM and RR tests, we found that rmTBI mice showed significant impairments in learning and memory function without accompanying persistent impairment of motor coordination; however, the Lip-1 and MSCs groups were remarkably superior to the vehicle group in persistent learning and memory function. Furthermore, we also observed increased neuronal degeneration and loss within the cortices and hippocampus in rmTBI mice, whereas treatment with MSC and Lip-1 significantly rescued neuronal degeneration and loss. Neuronal degeneration was negatively correlated with GPx activity. Therefore, we speculated that cognitive impairment and neurodegeneration in rmTBI mice are closely related to ferroptosis, and the therapeutic effect of MSCs can also be achieved by inhibiting ferroptosis after rmTBI. However, more studies are needed to explore the precise pathway of ferroptosis involved in impaired cognition after rmTBI.

AD is a common neurodegenerative disease after rmTBI and has similar characteristics to ferroptosis, such as iron overload, lipid peroxidation, and GPx4 inactivation [13]. Tau hyperphosphorylation, resulting in a neurofibrillary tangle, as well as the eventual axonal dysfunction, has been commonly observed among AD cases. Interestingly, tau hyperphosphorylation may result in ferroptosis [54]; however, the underlying mechanism is unclear. Another pathological feature of AD is Aβ deposition. A study found that hUCB-MSC co-culture with BV2 microglial cells treated with Aβ1–42 decreased Aβ within the medium, which was related to up-regulation of Aβ-degrading enzyme neprilysin within microglial cells [55]. According to our results, APP, Aβ, and Tau protein hyperphosphorylation levels increased after rmTBI, while levels decreased significantly in the MSC and Lip-1 groups compared with the vehicle group. Furthermore, the expression of Tau in the brain was analyzed by small animal PET/CT, and Tau levels within the cortices and hippocampi decreased in the MSCs and Lip-1 groups compared with the vehicle group. These results suggest that MSC treatment reduced pathological protein accumulation. Therefore, future studies will be needed to explore the mechanism of ferroptosis with additional pathological features to target anti-neurodegenerative disorder treatment.

Based on our findings, ferroptosis participates in the persistent cognitive impairment disease process of rmTBI; therefore, inhibiting ferroptosis may be an effective target to improve cognitive function after rmTBI. Taking into account the different mechanisms of ferroptosis, promising therapies targeting ferroptosis pathways could exhibit antioxidant or iron chelating characteristics [13]. This study suggested that MSCs exhibited antioxidant and iron chelating activities within rmTBI mice, similar to the ferroptosis inhibitor Lip-1. Therefore, this study suggested that MSCs suppress ferroptosis, demonstrating the potential role of MSCs as a ferroptosis inhibitor. This result has been confirmed by two latest studies [45, 56]. Our research found that MSCs may play a neuroprotective role partly by inhibiting ferroptosis. Previous studies have reported that MSCs can inhibit neuroinflammation and promote brain remodeling in TBI [57]. We will explore whether the inhibitory effect of MSCs on ferroptosis after rmTBI will affect other neuropathology using GPx4 knockout mice (ferroptosis over-activated) in future studies. However, while ferroptosis emerges as a critical cell death form in rmTBI, other programmed cell death pathways, including apoptosis, pyroptosis, necroptosis, and autophagy will be also activated after brain injury and determine the neuropathology as well as the cognitive prognosis [58–60]. The results of this research suggested that MSCs exert a neuroprotective effect by possibly inhibiting ferroptosis but also possible, by manipulating other cell death pathways. The purpose of this research is to provide a novel therapeutic strategy using MSCs transplantation to improve the cognitive outcome of rmTBI. More research is needed to explore which specific modes of cell death predominate in the pathological progression of rmTBI, which will lay the foundation for the clinical transformation of MSCs. Many studies have found that a combination treatment using inhibitors for various cell death pathways exhibited higher protective effects on neuroinflammation and neurological outcome than targeting a single pathway [13]. More research on combination strategies for the treatment of rmTBI is urgently needed.

The limitation of this study is that the optimal dose and frequency of MSC administration to reduce cognitive impairment of rmTBI, as well as the half-life and distribution of MSCs in vivo were not studied. Preclinical studies are needed to further explore the optimal dose and frequency of MSC administration, as well as the distribution and metabolism of MSCs. In addition, future studies will also need to focus on whether rmTBI results in peripheral dysfunction, and whether MSCs can eventually enter other tissues (immune tissue, liver, lung, gut, etc.) to mitigate the secondary effects of rmTBI. Furthermore, only male mice were used in this research, and female mice were not involved. Therefore, the gender differences of rmTBI and the therapeutic effects of MSC in mice of different sex have not been elucidated. Follow-up studies in female mice should be supplemented to reduce experimental bias caused by gender factors. Moreover, our research found that RR did not show differences in motor function. RR may not be sensitive enough to detect smaller changes in locomotor function, and further studies could consider using more test methods (e.g., catwalk) for validation.

## Conclusions

To summarize, this study demonstrated the presence of ferroptosis after rmTBI, characterized by iron accumulation, abnormal GPx4, increased lipid peroxidation, and shrunken mitochondria. In addition, MSCs could reduce neuronal degeneration and loss, decrease pathological protein deposition, and ameliorate persistent cognitive impairment of rmTBI, possibly by inhibiting ferroptosis. We believe that the results of this study provide insight on post-rmTBI cell death and lay the fundamental basis for future research on cell death targeting therapies for rmTBI.

## Data Availability

The datasets used and analyzed during the current study are available from the corresponding author on reasonable request.

## Abbreviations

rmTBI: Repetitive mild traumatic brain injury
mTBI: Mild traumatic brain injury
MSCs: Mesenchymal stromal cells
GPx: Glutathione peroxidase
PD: Parkinson's disease
AD: Alzheimer’ s disease
ROS: Reactive oxygen species
GPx4: Glutathione peroxidase 4
CNS: Central nervous system
Tfr1: Transferrin receptor-1
Fpn: Ferroportin
4-HNE: 4-Hydroxynonenal
SCs: Stem cells
hUCB: Human umbilical cord blood
WB: Western blot
TEM: Transmission electron microscopy
FJC: Fluoro-Jade C
IF: Immunofluorescence
NOR: Novel object recognition
MWM: Morris water maze
PET/CT: Positron emission tomography/computed tomography
NORI: Novel object recognition index
SPT: Spatial probe test
FDG: ^18^F-fluoro-2-deoxy-d-glucose
FOV: Field of view
PFA: Paraformaldehyde
PBS: Phosphate-buffered saline
DAPI: 6-Diamidino-2-phenylindole
SDS-PAGE: Sodium dodecyl sulfate-polyacrylamide gel electrophoresis
APP: Amyloid precursor protein
Aβ: Beta-amyloid
p-Tau: Phospho-tau
SD: Standard deviation
ANOVA: Analysis of variance
BBB: Blood–brain barrier
SUV: Standardized uptake value
Tf: Transferrin
Tf–Tfr: Tf–transferrin receptor
